# A simple and inexpensive quantitative technique for determining chemical sensitivity in *Saccharomyces cerevisiae*

**DOI:** 10.1038/s41598-018-30305-z

**Published:** 2018-08-09

**Authors:** Chao-Wei Hung, Jorge Y. Martínez-Márquez, Fatima T. Javed, Mara C. Duncan

**Affiliations:** 1 Department of Biology, University of North Carolina, Chapel Hill, North Carolina, USA; 2 Department of Cell and Developmental Biology, University of Michigan, Ann Arbor, Michigan, USA; 3 Present Address: Department of Medicine, University of California, San Diego, California, USA

## Abstract

Chemical sensitivity, growth inhibition in response to a chemical, is a powerful phenotype that can reveal insight into diverse cellular processes. Chemical sensitivity assays are used in nearly every model system, however the yeast *Saccharomyces cerevisiae* provides a particularly powerful platform for discovery and mechanistic insight from chemical sensitivity assays. Here we describe a simple and inexpensive approach to determine chemical sensitivity quantitatively in yeast in the form of half maximal inhibitory concentration (IC_50_) using common laboratory equipment. We demonstrate the utility of this method using chemicals commonly used to monitor changes in membrane traffic. When compared to traditional agar-based plating methods, this method is more sensitive and can detect defects not apparent using other protocols. Additionally, this method reduces the experimental protocol from five days to 18 hours for the toxic amino acid canavanine. Furthermore, this method provides reliable results using lower amounts of chemicals. Finally, this method is easily adapted to additional chemicals as demonstrated with an engineered system that activates the spindle assembly checkpoint in response to rapamycin with differing efficiencies. This approach provides researchers with a cost-effective method to perform chemical genetic profiling without specialized equipment.

## Introduction

Chemical sensitivity is a powerful phenotype that provides an easy avenue to identify and characterize genes important for diverse cellular processes. Although growth-based chemical sensitivity assays are used in nearly every model system, yeast provides a particularly powerful platform for discovery and mechanistic insight from chemical sensitivity assays because of the ease of culturing and analysis. Indeed, chemical sensitivity assays in yeast have provided seminal insight into the function of diverse protein families^1–3^. An important aspect of growth-based chemical sensitivity assays is that when performed correctly, it produces a quantitative read-out.

A quantitative read-out is desirable for many reasons. Perhaps most importantly, a quantitative read-out is a solid foundation for unbiased, rigorous, and statistically significant findings. Furthermore, a quantitative read-out is a required basis for informatics analyses. Indeed, recent studies used quantitative growth based chemical sensitivity combined with clustering analysis to deduce the function of different genes and the mechanisms of bioactive chemicals^4–6^.

Unfortunately, published quantitative chemical sensitivity growth based protocols describe techniques using expensive equipment or technology. These technically advanced assays rely on deep sequencing or microarray to monitor changes in population over time, which are difficult for the average lab to implement, or use more expensive rotary shaking reader or robotic systems^5,7–13^. Consequently, most laboratories without expertise in chemical screening in yeast rely on published plating assays to monitor chemical sensitivity, referred to as the “traditional plating assay” throughout this paper. In this method, cells of different genotypes are plated onto agar plates supplemented with a chemical. Sensitivity or resistance is then determined by monitoring presence or absence of colonies, the size of colony formed, and/or the number of colonies formed.

Although the traditional plating assay is accessible to laboratories with limited resources, this method has several drawbacks. First, the read-outs from the traditional plating assay are poorly quantitative. The effect of a chemical on colony forming units can be affected by minor errors in dilution or plating. Similarly, the use of colony size as a measure of effect is affected by nutrient depletion by rapidly growing colonies, which can result in a plateau in colony size. This plateau allows slower growing colonies to “catch up’” to more rapidly growing colonies and thus, unless imaged at the correct time, growth difference can be hard to quantitate. Furthermore, analysis of growth differences can be complicated if a mutation alters cell growth rate independent of chemical treatment. Second, each agar plate can only test for a single chemical concentration. Therefore, in order to identify the optimal chemical concentrations for an assay, multiple plates are required and each plate can require a large amount of chemical. These factors can make chemical sensitivity assays costly and time consuming. Furthermore, in the traditional plating assay, single cells must form colonies, which even under normal conditions takes two days at 30 °C or four days at room temperature. Since many chemicals have limited life-spans under such conditions, this limits the utility of such assays. Finally, the technical challenges inherent in preparing the chemical plates can lead to variability between experiments. Chemicals are frequently added to molten agar. Differences in the temperature of the agar when the chemical is added may change the specific activity of chemicals in plates from different batches. Alternatively, top-spreading the chemical, which avoids the problems from molten agar, can result in uneven chemical distribution or changes in chemical distribution over time as the chemical diffuses from the top of the plate into the depth of the agar. These limitations reduce the utility of this otherwise highly powerful class of phenotypes using the traditional plating method.

To overcome the limitation of the traditional plating assay, several groups have published protocols for monitoring yeast growth in 96-well plates using advanced plate readers with incubating and multi-modal shaking capabilities^7,14^. However, many labs, including our own, perform chemical sensitivity assays with standard plate readers^15,16^. To date, no comprehensive description of how to adapt a plating assay to a plate reader assay has been provided. Here we provide a roadmap for the non-expert lab to perform simple, inexpensive chemical sensitivity assays in liquid media in 96-well plates. This method provides several improvements over the commonly used traditional plating assays. By using smaller volumes, this method dramatically reduces the costs of the analysis. It reduces the incubation time, thereby increasing through-put and reducing the risks of chemical inactivation. It uses simple, readily available tools, making this protocol suitable for most research environments, including undergraduate teaching laboratories. Importantly, this method allows for a reproducible quantitative measure in the form of half maximal inhibitory concentration (IC_50_). Furthermore, by adding chemicals to liquid media at room-temperature, it overcomes variability problems inherent in the traditional plating method. Finally, unlike previously published 96-well plate growth assays, this method does not require a plate reader capable of multimodal shaking, or robotic sample readers, and can be adapted to end-point assays compatible with a standard spectrometer. We describe steps needed to overcome some of the common problems encountered when performing chemical sensitivity assays in 96-well plates without multimodal shaking, how to detect these problems, and solutions. Together these features provide an inexpensive, easily implemented approach to quantitatively monitor chemical sensitivity in yeast.

As a proof of principle, we applied this method to four chemicals commonly used to assess defects in membrane traffic in yeast. We show that the liquid based assay reveals roles for clathrin adaptors at the *trans-*Golgi Network (TGN) and endosomes in the traffic of the chitin synthase Chs3 that are not apparent from the traditional plating method using the cell wall binding toxin calcofluor white (CFW). We also show that the liquid based assay substantially reduced the number of days required to observe changes in sensitivity to the toxic amino acid analog canavanine. Additionally, we showed this liquid assay significantly reduced the amount of chemical required to assay the effect of myriocin and sertraline on cell growth. Finally, we demonstrate the general applicability of this system by monitoring quantitative differences in checkpoint activation using an engineered chemical-induced dimerization system.

## Results and Discussion

### Adapting Calcofluor White to a 96-well plate assay

In the yeast *Saccharomyces cerevisiae*, defects in clathrin mediated traffic at the TGN and endosomes alters sensitivity to CFW. CFW is a cell wall binding toxin thought to kill cells by disrupting cell wall integrity^17,18^. Screens for genes important for CFW sensitivity revealed that the chitin synthase Chs3 is required for CFW lethality, as are a complex of proteins known as exomer, which delivers Chs3 to the plasma membrane^19^. Cells lacking the exomer subunit Chs6 are resistant to CFW, because Chs3 is trapped at the TGN and endosomes^20^. This intracellular retention of Chs3 in *chs6*Δ cells requires clathrin mediated traffic at the TGN and endosomes. Therefore, mutations that disrupt traffic at the TGN and endosomes can restore plasma membrane localization of Chs3 in *chs6*Δ cells, rendering *chs6*Δ cells sensitive to CFW^21^. This restoration of CFW sensitivity to *chs6*Δ cells has been used to classify mutations and chemicals as causing defects in TGN sorting^15,22–25^.

The yeast epsin-like proteins Ent3 and Ent5 were previously reported to perform partially redundant functions in retaining Chs3 at the TGN and endosomes based on the traditional plating assay^23^. This redundancy was suggested by findings that combined deletion of *ENT5* and *ENT3* suppressed the CFW resistance of *chs6*Δ cells, whereas deletion of only one gene did not suppress the resistance^23^. We sought to use the CFW sensitivity as a method to quantify defects in *ENT5* caused by a series of point mutations that disrupted Ent5 interaction with lipids (CR), clathrin (ΔCB), adaptor proteins that contain γ ear domain (ΔAB) and both adaptors and clathrin (ΔABCB). In other assays of Ent5 function, these alleles show different levels of impairment^15^.

**Figure 1:**
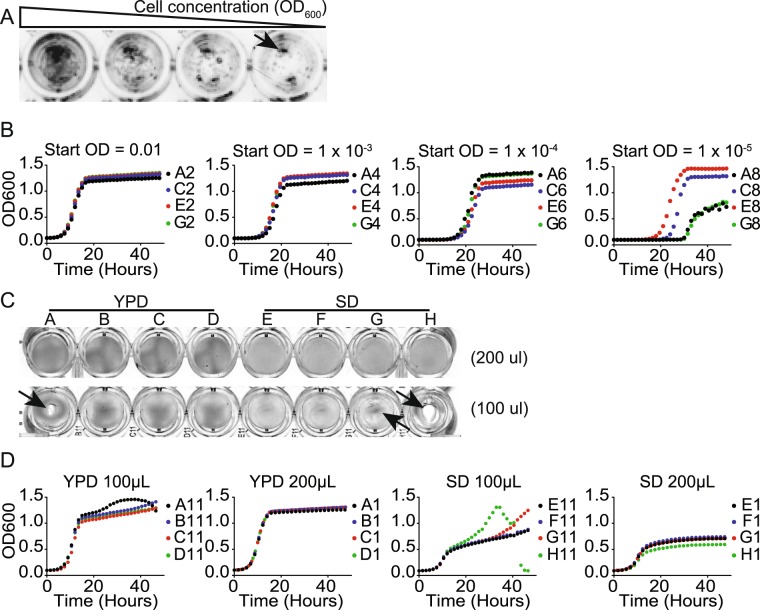
Optimization of growth curve experiments. (**A**) The effect of initial cell concentration on the formation of micro-colonies after 24 hours of incubation in YPD. The arrow indicates micro-colonies. Image is shown with black and white inverted. (**B**) The effect of initial cell concentration on the reproducibility of growth curves. Indicated optical densities (OD_600nm_) of starting cultures were grown in YPD. OD_600nm_ was measured every 30 minutes. Each growth curve represents a technical replicate. (**C**) The effect of the volume of media on the clumping of cells and (**D**) The reproducibility of growth curves; labels represent the positions on a 96-well plate. Each curve growth represents a technical replicate with 0.01of starting OD_600nm_, grown in indicated amount of YPD of SD media, measured every 30 minutes.

We initially followed established protocols for 96-well based growth assays in liquid cultures. The published assays called for nearly continuous agitation of the plate^26–28^. However, we found that under these conditions cells clumped in the middle and sides of the wells giving variable reading as previously reported by some (data not shown)^29^. We next attempted intermittent agitation, which is reported to reduce variability^29^. However, like the continuous agitation, we found the cells accumulated in clumps leading to variable readings. Finally, we attempted to monitor growth without any agitation. We found that when we used a low number of viable cells per well as generally used for agitated cultures, individual colonies formed on the bottom of the well leading to variable readings (Fig. 1A,B). However, if we started with a higher number of cells, a uniform lawn formed leading to highly reproducible values (Fig. 1B). We found the most reproducible results came from an initial starting OD_600_ of 0.01 using 200 μL of media. Lower media volumes lead to variability due to cell clumping (Fig. 1C,D arrows). These conditions worked well for both YPD and SD, two commonly used yeast media.

**Figure 2:**
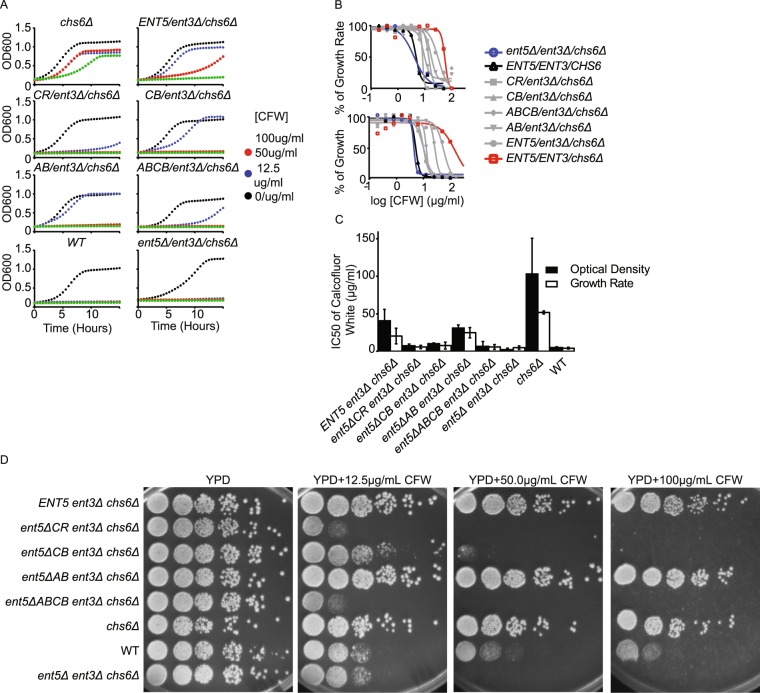
Developing a quantitative CFW assay. (**A**) Indicated strains were grown to log phase and transferred to 96 well plates. Indicated concentrations of CFW were added and OD was monitored every 30 minutes for 19.5 hr. (**B**) Dose dependence curves of indicated strains determined from growth-rate during logarithmic phase (top) and OD at 19.5 hrs. (**C**) IC50 values determined from OD at 19.5 hrs (black) and growth rate (white). (**D**) Indicated strains were grown to log phase, serially diluted and replica pinned onto YPD or YPD containing indicated concentration of CFW. Plates were cultured at 30 C for 3 days. Statistical analysis shown in Table 3.

Using these culturing conditions, we then established conditions for CFW sensitivity. To do this, we grew wild-type and mutant cells in a 96-well plate in YPD media supplemented with different concentrations of CFW and assayed the cell growth by measuring the optical density (OD) every 30 minutes for 24 hours (Fig. 2A, see materials and methods). The highest concentration of CFW we used in our study was 100 μg/mL of CFW. This concentration only slightly affected the growth of *chs6*Δ cells. However, this concentration completely inhibited the growth of *ent3Δ ent5Δ chs6Δ* cells as previously reported for the traditional plating assay (Fig. 2A,D), establishing the effectiveness of the simple inexpensive assay to monitor known sensitivities.

We next used the liquid assay to quantify defects in *ENT5* caused by point mutations that are known to disrupt different biochemical activities of Ent5^15^. To determine IC_50_ of CFW, we first generated the dose dependent curve by plotting the growth rate (the slope of exponential growth phase) normalized to that of the untreated cells as the function of CFW concentrations in log (Fig. 2B, top). We then used Graphpad Prism to fit the dose dependent curve by using the following sigmoidal equation:

$$y=bottom+\frac{{\rm{top}}-{\rm{bottom}}}{1+{10}^{(\mathrm{log}1{\rm{c}}50-{\rm{x}})\ast {\rm{H}}}},$$

where top is the maximal growth rate for a strain, bottom is the minimal growth rate and H is the Hill slope. Although the growth rate is a commonly accepted parameter for determining chemical sensitivity^30^, we had to exclude data from several concentrations in our calculation because cells showed little growth, thus the growth rate was difficult to determine. The exclusion of this data obscured important differences in sensitivity. For example, IC_50_ calculated by growth rate suggested that *ent3*Δ *ent5*Δ *chs6*Δ (IC_50_ = 4.84 µg/mL) and *ent5ΔABCB ent3Δ chs6Δ* ((IC_50_ = 5.78 µg/mL) are equally affected by CFW. However, it is clear from the individual growth curves that *ent3*Δ *ent5*Δ *chs6*Δ is more sensitive that *ent5ΔABCB ent3Δ chs6Δ* (Fig. 2A). Therefore, to solve this problem, we generated the dose dependent curve by first determining the time-point when wild type, untreated cells exit the exponential growth phase. We then used the OD for all samples at that time point normalized to the maximum OD of untreated cells to determine a dose response curve (Fig. 2B, bottom) and determine the IC_50_ using the sigmoidal equation as described above (Fig. 2C, Table 1). We also performed statistical analysis to show that results are statistically significant (Table 2).

**Table 1:** List of IC50 values and standard deviation determined by OD (OD), growth rate (GR) and end-point assays (EP) as described in Fig. 2 (CFW), Fig. 3 (Can), Fig. 4 (Myriocin), Fig. 5 (Sertraline) and Fig. 6 (Rapamycin).

	CFW (μg/ml)	Can (μg/ml)	Myriocin (nM)	Sertaline (nM)	Rapa (ng/ml)
WT	4.98 ± 1.09, n = 3 (OD) 4.15 ± 1.21, n = 3 (GR)	1.5 ± 0.28, n = 6 1.83 ± 0.04. n = 2 (EP)	565 ± 140, n = 16	11.61 ± 1.00, n = 4	0.20 ± 0.0036, n = 4
*ent3Δ chs6Δ*	40.72 ± 15.23, n = 3 (OD) 20.27 ± 10.41, n = 3 (GR)				
*ent5ΔCR ent3Δ chs6Δ*	7.37 ± 2.23, n = 3 (OD) 5.75 ± 2.25, n = 3 (GR)				
*ent5ΔCB ent3Δ chs6Δ*	10.12 ± 1.056, n = 3 (OD) 7.63 ± 4.63, n = 3 (GR)				
*ent5ΔAB ent3Δ chs6Δ*	31.04 ± 4.11, n = 3 (OD) 24.86 ± 6.84, n = 3 (GR)				
*ent5ΔABCB ent3Δ chs6Δ*	6.61 ± 6.48, n = 3 (OD) 5.78 ± 3.15, n = 3 (GR)				
*ent5Δ ent3Δ chs6Δ*	2.28 ± 1.89, n = 3 (OD) 4.84 ± 2.24, n = 3 (GR)				
*chs6Δ*	103.2 ± 47.53, n = 3 (OD) 51.79 ± 2.03, n = 3 (GR)				
*art1Δ*		0.50 ± 0.13, n = 6 0.28 ± 0.01, n = 2 (EP)			
*apm1Δ*			367 ± 150, n = 16	12.96 ± 1.4, n = 4	
*apm2Δ*			420 ± 140, n = 16	6.57 ± 0.4, n = 4	
*ASK tor1-1*					27.6 ± 0.8, n = 4
*DAD2 tor1-1*					5.5 ± 0.8, n = 4
*MTW1 tor1-1*					0.04 ± 0.01, n = 4

**Table 2:** Statistical Analysis for Fig. 2C. Two-tailed P values are calculated by comparing the indicated strains to *ent3Δchs6Δ*.

ent5ΔCR ent3Δ chs6Δ	0.0039
ent5ΔCB ent3Δ chs6Δ	0.0128
ent5ΔAB ent3Δ chs6Δ	0.0656
ent5ΔABCB ent3Δ chs6Δ	0.003
ent5Δ ent3Δ chs6Δ	0.0106
chs6Δ	0.0427
WT	0.133

Using this OD method, we discovered that deletion of *ENT3* reduces the IC_50_ of CFW of *chs6*Δ cells by about 2-fold (Fig. 2C, Table 1). This indicates that, contrary to prior reports, Ent3 may have a non-redundant role in the traffic of Chs3 at the TGN and endosomes. Moreover, we found all mutations of *ENT5* tested further reduced the IC_50_ of CFW of *ent3Δchs6Δ* cells. The magnitudes of the effects were consistent with the overall effects of these mutations on Ent5 function in that *ent5ΔA*B, which has the weakest effect on Ent5 function, was the least sensitive to CFW whereas *ent5ΔCR*, which has the strongest effect on Ent5 function, had an IC_50_ similar to the null allele^15^. These results establish that this simple inexpensive assay can effectively quantify growth sensitivities without the need for specialized equipment.

We compared these results to those obtained with the traditional plating assay. Using the same concentrations, the traditional plating assay could detect the increase sensitivity of most alleles. However, it was unable to detect the increased sensitivity of the *ent5ΔAB* allele or the *ent3Δ* (Fig. 2D). Notably, the *ent3*Δ *chs6*Δ strain barely grew in the presence of 100 µg/mL CFW in the liquid assay (Fig. 2A), whereas these cells grow robustly on the same concentration of CFW in the traditional plating assay (Fig. 2D). Similarly, in the liquid culture 50 µg/mL CFW was sufficient to strongly inhibit the growth of *ent5ΔAB ent3Δ chs6Δ*, whereas no detectable effect was observed on agar plates supplemented with 100 µg/mL of CFW (compare Fig. 2A,D). We hypothesize that the increased sensitivity of yeast to CFW in the liquid assay may be due to the differences inherent in exposing cells to chemicals in liquid versus on a plate. In liquid media, cells are bathed in the chemical, thus the concentration encountered by each cell is likely equal to the concentration of the chemical in the liquid media for most cells. In contrast, when cells are grown on a solid agar plate supplemented with chemicals, only cells close to the surface of the agar plate are exposed to chemical, and the concentration at the surface of the plate may be substantially less than the concentration in the plate. Therefore, the liquid based method may produce a more accurate assessment of the actual inhibitory effect of a chemical than the traditional plating method.

### Adapting Toxic Amino Acid Analogs to a 96-well plate assay

**Figure 3:**
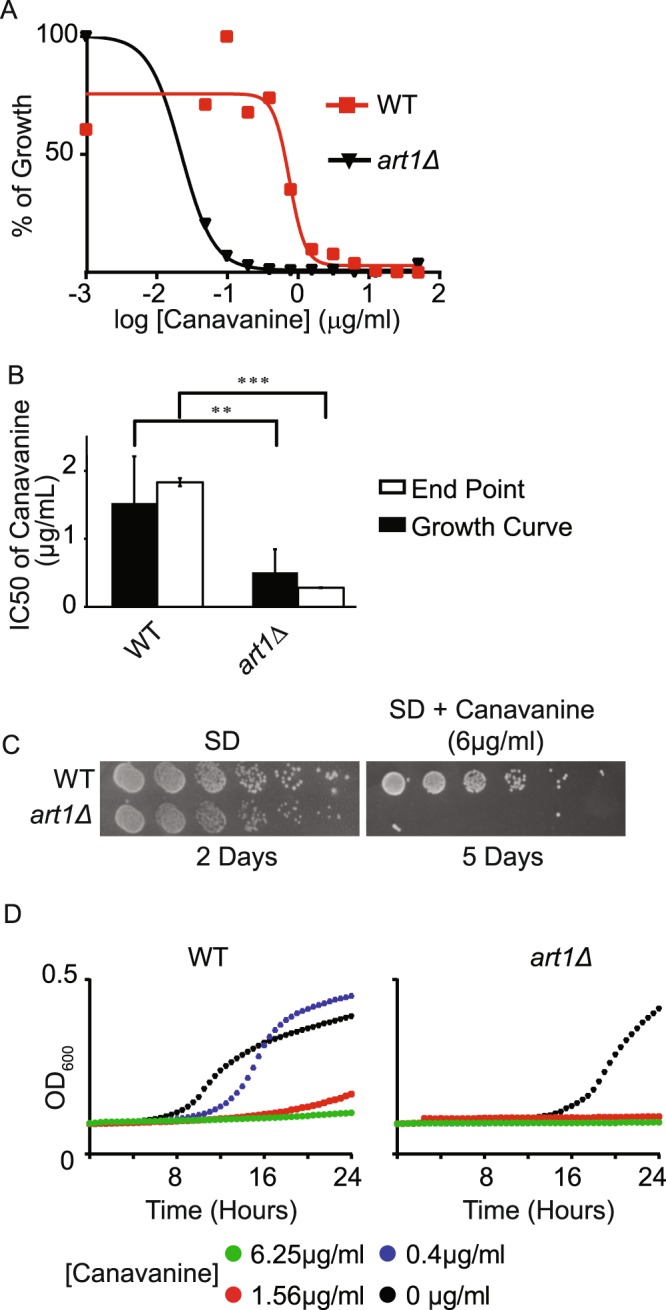
Developing a quantitative canavanine assay. (**A**) Dose dependence curves of indicated strains determined from OD at 20 hrs. (**B**) IC50 values determined from OD at 20 hrs or an endpoint assay from plates cultured in an incubator for 18 hrs. (**C**) Indicated strains were grown to log phase and serially diluted by 2-fold. Dilutions were replica pinned onto a SD or SD agar plate supplemented with indicated concentration of canavanine. (**D**) Indicated strains were grown to log phase and transferred to 96 well plates. Indicated concentrations of canavanine were added and OD was monitored every 15 minutes for 24 hr. **p < 0.01, ***p < 0.001.

Like CFW, toxic amino acid analogs are commonly used to monitor membrane traffic in yeast^31–34^. The basis of these assays is that toxic amino acids enter cells through amino acid permeases. Therefore, changes in the amount of amino acid permeases at the cell surface caused by defects in membrane traffic changes the sensitivity of cells to toxic amino acids that enter the cell through that permease. Indeed, a genome wide screen for sensitivity to the toxic amino acid canavanine identified the yeast alpha-arrestin, Ldb19/Art1 as an important modulator of the endocytosis of the arginine permease Can1^3^. We established conditions for canavanine sensitivity in the liquid assay. As with the CFW assay, we used the concentration of canavanine established in the traditional plating assay as our maximal concentration and determined IC_50_ values. We found Art1 was three times more sensitive to canavanine than wild type cells (Fig. 3A,B, Table 1).

We noted several advantages for the liquid assay compared to the traditional plating assay for canavanine sensitivity. One major challenge with using toxic amino acids is that wild-type cells internalize toxic amino acids and grow more slowly in their presence. Thus, at the concentrations required to reveal sensitivity of *art1Δ* to canavanine, wild-type cells must grow for 5 days for colonies to grow to sufficient size for an accurate assessment of growth defects (Fig. 3C). In contrast, the liquid media-based approach, we saw a difference in canavanine sensitivity between wild-type and *art1Δ* cells within 24 hours (Fig. 3D). Thus, the liquid media approach is more time efficient than the traditional plating assay. Furthermore, an added advantage of the liquid media approach is that it can accurately compensate for the differences in growth rate caused by genotype. *art1Δ* grows more slowly than wild-type cells on plates and in liquid media. By normalizing to the growth of untreated *art1Δ* cells, the liquid assay can accurately compensate for this difference. Using this approach, we determined that *art1Δ* cells are three times more sensitive to canavanine than wild-type cells (Fig. 3B, Table 1). This phenotype is consistent with the known effect of Art1 on the endocytosis of Can1^3^.

One issue with the method as described is that each assay occupies the plate reader for an entire day. This limits the number of strains and conditions that can be assayed at one time. We asked whether we could increase through-put by growing plates outside of the plate reader and measuring absorbance at a single time-point. To do this, we used the growth curves from plates grown in the plate-reader to determine the optimum time-point for an ‘end-point’ read. We found that the optimum time-point is the time at which wild-type untreated cells exit logarithmic phase (18 hours for canavanine conditions). This time point is optimum because it allows slow growing cultures the most time to grow and produce detectable increases in OD, while preventing fast growing cultures from oversaturating which would lead to an underestimation of growth. We then prepared 96-well plates exactly as for the plate-reader assay but incubated them in a humid chamber inside a standard incubator. At 18 hours, we measured the end-point OD. We found that the IC_50_ of wild type and *art1Δ* determined in this way was not significantly different from that determined from growth within the plate reader (Fig. 3D, Table 1). Thus, the endpoint assay provides an easy low-cost method to determine IC_50_ values for a large number of strains at the same time.

### Adapting Sertraline and Myriocin to a 96-well plate assay

Myriocin and Sertraline have recently been reported to distinguish between different trafficking pathways at the TGN and endosomes^35^. Myriocin is a well-studied inhibitor of sphingolipid biosynthesis. It targets serine palmitoyltransferase, the enzyme that catalyzes the first step of biosynthesis of sphingolipids^36,37^. Sertraline is a commercially available antidepressant that has been reported to inhibit yeast growth^38^. In the traditional plating assay, sensitivity to these two compounds distinguishes between cells lacking *APM1* or *APM2*, which encode two alternate μ subunits for the clathrin adaptor protein complex-1 (AP-1)^35^. Cells lacking *APM1* are more sensitive to myriocin than wild-type cells using the traditional plating assay. In contrast, cells lacking *APM2* are as sensitive to wild-type cells using the traditional plating assay. Similarly, cells lacking *APM1* and *APM2* have different effects in sertraline sensitivity. Cells lacking *APM1* are resistant to sertraline whereas cells lacking *APM2* are more sensitive to sertraline than wild-type cells. Thus, based on the traditional plating assay these two chemicals can selectively detect defects in *APM1* and *APM2* dependent trafficking.

**Figure 4:**
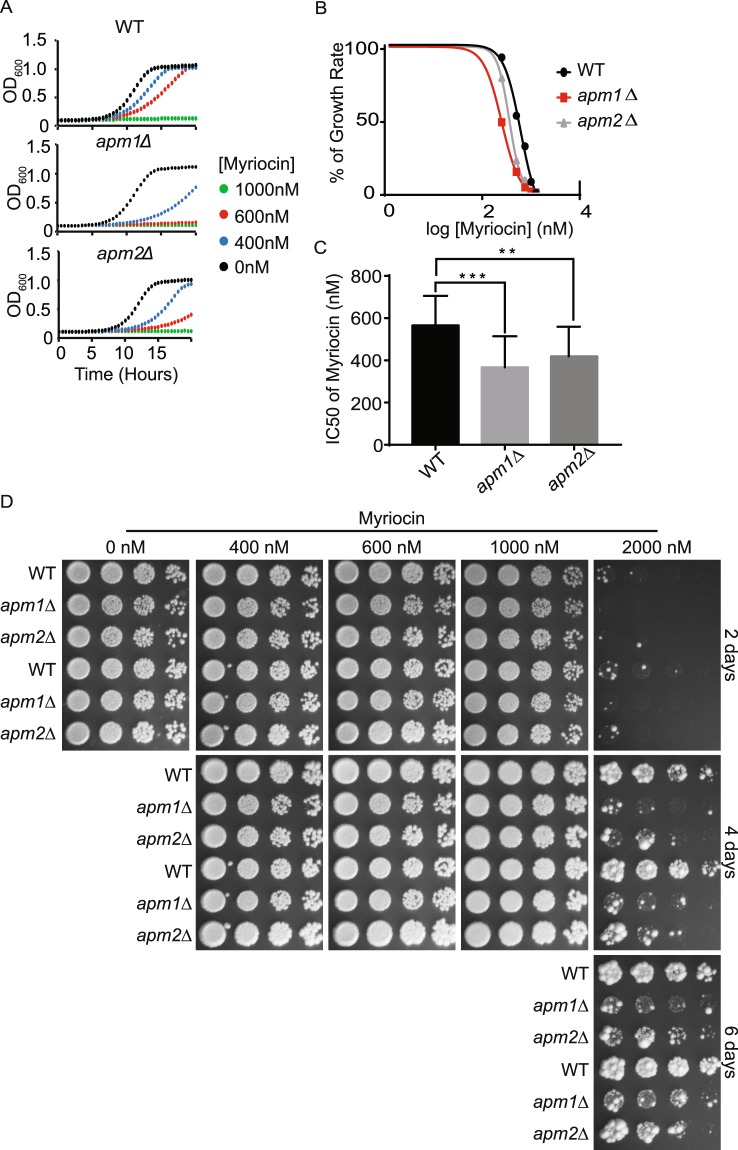
Developing a quantitative myriocin assay. (**A**) Indicated strains were grown to log phase and transferred to 96 well plates. Indicated concentrations of myriocin were added and OD was monitored every 15 minutes for 24 hr. (**B**) Dose dependence curves of indicated strains determined from OD at 20 hrs in the presence of myriocin. (**C**) IC50 values to sertraline determined from OD at 20 hrs. (**D**) Indicated strains were grown to log phase, serially diluted and replica pinned onto YPD or YPD containing indicated concentration of CFW. Plates were cultured at 30 C and imaged after 2, 4 and 6 days. **p < 0.01, ***p < 0.0005.

**Figure 5:**
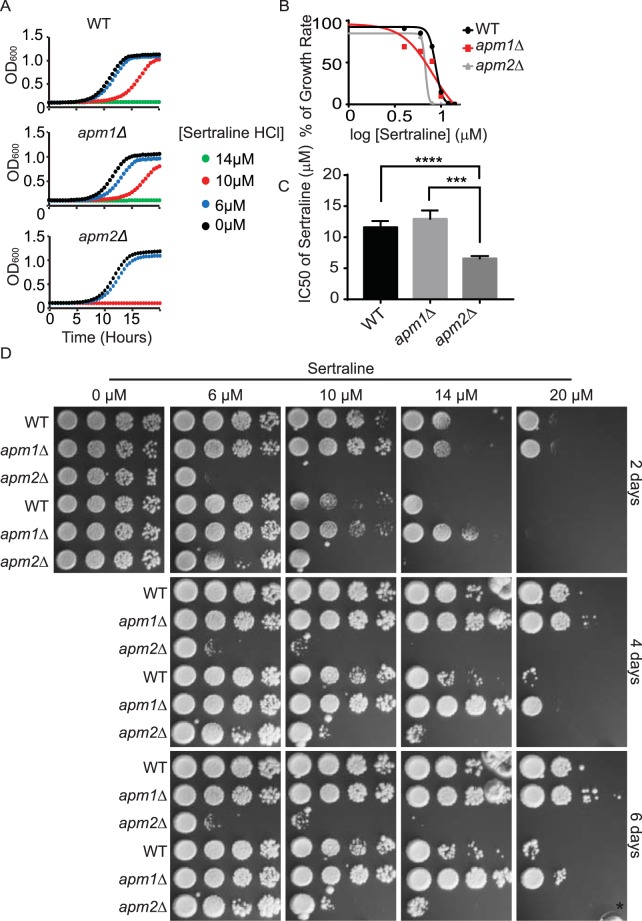
Developing a quantitative sertraline assay. (**A**) Indicated strains were grown to log phase and transferred to 96 well plates. Indicated concentrations of sertraline were added and OD was monitored every 15 minutes for 24 hr. (**B**) Dose dependence curves of indicated strains determined from OD at 20 hrs in the presence of sertraline. (**C**) IC50 values to sertraline determined from OD at 20 hrs. (**D**) Indicated strains were grown to log phase, serially diluted and replica pinned onto YPD or YPD containing indicated concentration of CFW. Plates were cultured at 30C and imaged after 2, 4 and 6 days. *Indicates fungal contamination ****p < 0.0001, ***p < 0.001.

To expand the tool-kit of quantitative trafficking assays, we determined the conditions for both myriocin and sertraline in the 96-well assay. We found that in the 96-well assay, both myriocin and sertraline completely inhibited the growth of wild-type cells at lower concentrations than are needed in the traditional plating assay^35^. For myriocin, 1 μM was sufficient to completely inhibit cell growth for 18 hours (Fig. 4A,B), whereas in the traditional plating assay, wild-type cells grew in the presence of 1 μM myriocin (Fig. 4D). Similarly, wild-type cells were completely inhibited by 6 μM of sertraline in the liquid assay (Fig. 5A,B) whereas in the traditional plating assay wild-type cells were able to grow well in 10 μM sertraline (Fig. 5D). When we monitored the effects of myriocin and sertraline on cells lacking *APM1* and *APM2* in the liquid assay, we saw similar but not identical results to the effects in the traditional plating assay. As previously reported, cells lacking *APM1* were sensitive to myriocin and cells lacking *APM2* were sensitive to sertraline in the liquid assay. However, in contrast to previous reports, cells lacking *APM2* were also sensitive to myriocin in the liquid assay (Fig. 4C). In fact, when incubated at high concentration of myriocin (2 μM) for at least 4 days, the traditional plating assay was able to reveal that cells lacking *APM2* are also sensitive to myriocin (Fig. 4D). This result demonstrates that the liquid assay is able to detect cell growth defects at lower drug concentrations and in a shorter period of time. More surprisingly however, we were unable to detect resistance of cells lacking *APM1* to sertraline by liquid assay (Fig. 5C). In contrast, the traditional plating assay was able to detect the resistance to sertraline when incubating cells in the presence of 20 μM of sertraline for at least 4 days (Fig. 5D). This difference may be due to the extended incubation used for traditional plating assay. This extended growth incubation period may enhance minor improvements in growth rate that we are unable to detect after only 18 hours of growth. Thus, the short time-frame used for the 96-well assay, may not be suitable to detect resistance to a compound if the effect is small.

### Adapting rapamycin induced dimerization based sensitivity to a 96-well assay

We next sought to determine whether the conditions established for membrane trafficking assays could be applied to chemicals that act on completely unrelated pathways. To do this we selected, the chemical induced dimerization (CID) technique. CID is a powerful method to manipulate protein-protein interactions and activities^39^. The CID technique uses a cell permeant chemical to dimerize two proteins together. One of the most widely used chemical dimerizer is rapamycin. Rapamycin induces the dimerization of the FK506 binding protein (FKBP) and the FKBP12-rapamycin binding protein (FRB). In a rapamycin based CID system, one protein is genetically fused to FKBP and the other protein of interest is fused to FRB^40^. Using this approach, rapamycin based CID can be used to induce gene expression, protein-protein interactions, and protein translocation in live cells^41–43^.

**Figure 6:**
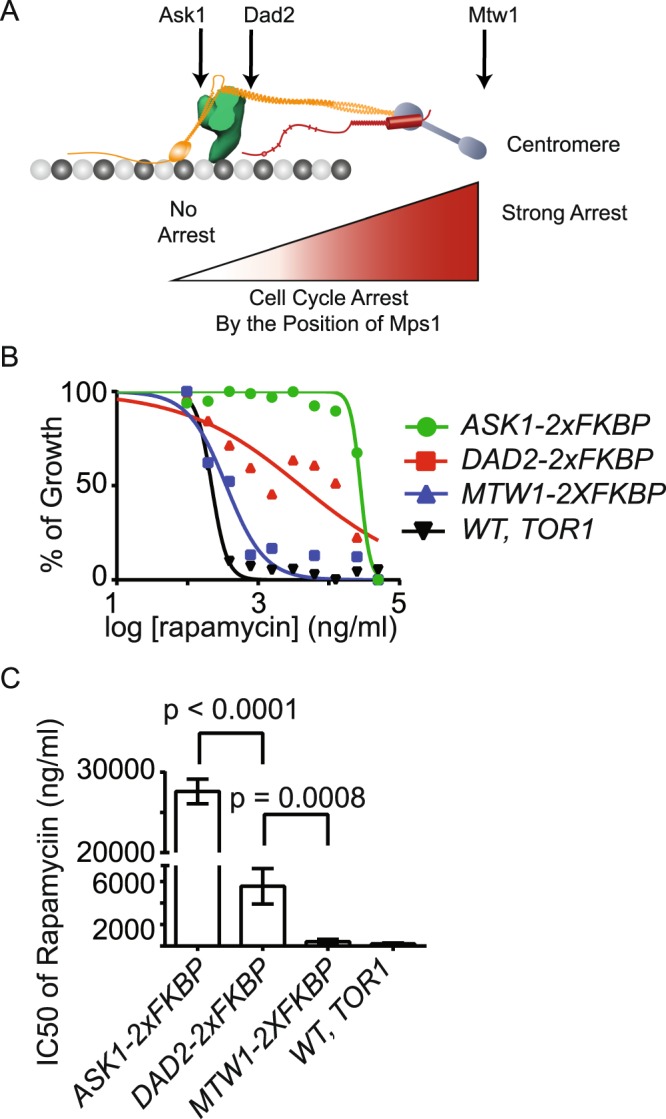
Developing a quantitative assay for inhibition due to CID. (**A**) Schematic of the yeast kinetochore. The locations of anchor proteins used are indicated (top) and strength of cell cycle arrest cause by anchoring Mps1 to each location is indicated by the gradient from red (complete arrest) to white (no arrest). (**B**) Dose dependence curves of strains containing *tor1-1, MPS1-FRB* and indicated anchor or wild-type control determined from OD at 20 hrs. (**C**) IC50 values determined from OD at 20 hrs.

Rapamycin is normally a potent inhibitor of yeast growth because it inhibits mTORC1, an essential kinase^44^. However, rapamycin can be effectively used for CID in yeast because of an available rapamycin resistant allele of *TOR1*, a kinase subunit of mTORC1^2,45^. Rapamycin based CID has been used to rapidly inhibit protein function in many studies^46–48^. In these cases, rapamycin sensitive growth could provide a read-out for whether the targeted protein is required for cell growth. However, rapamycin sensitive growth can be applied for many other uses. For example, it was used to probe the ability of the spindle assembly checkpoint (SAC) kinase Mps1 to activate the checkpoint from different locations within the kinetochore^49,50^. In this case, anchoring Mps1 to different locations within the kinetochore caused different levels of cell cycle arrest depending on the proximity of Mps1 to its substrate Spc105 (Fig. 6A). However, this position-specific activation led to an incomplete cell cycle arrest in some cases. This incomplete cell cycle arrest was difficult to accurately assess from colony size. Given the utility of rapamycin based CID to study a wide-variety of functions, a robust quantitative chemical sensitivity assay is important for rapamycin based CID. We therefore asked if the liquid based assay could provide a quantitative read-out of growth inhibition using rapamycin based CID.

To determine if the liquid culture method could be used to monitor defects in cell growth caused by rapamycin induced CID, we monitored the effects of rapamycin on the growth of cells with CID induced activation of the spindle assembly checkpoint. In this set-up, CID localization of the SAC activating kinase Mps1 to locations near the centromere causes strong checkpoint activation and inhibits growth, whereas localization further away from the centromere causes a weaker activation that slows cell division without inhibiting growth^50^. Consistent with previous reports based on the traditional plating method, when Mps1-FRB was anchored at Mtw1, an inner kinetochore component (near the centromere), cell growth was strongly inhibited (Fig. 6B,C). This strong inhibition is consistent with the previously reported constitutive activation of the checkpoint in this strain^50^. In contrast, when Mps1-FRB is anchored at Ask1, an outer kinetochore component (far from the centromere) cell growth was unaffected (Fig. 6B,C). This lack of inhibition is consistent with the previously reported normal cell cycle in this strain^50^. Finally, we found that cells where Mps1 was anchored at a point between the inner and outer kinetochore (Dad2), the cells grew at reduced rates and the cells were five times more sensitive to rapamycin than when Mps1 was anchored to Ask1 (Fig. 6B,C). This reduction is consistent with the previously reported cell cycle delay in this strain. Thus, the liquid culture method works for chemicals that reduce or inhibit cell growth by a wide variety of mechanisms and can be used to quantitate differences in growth caused by rapamycin induced CID.

## Discussion

The approach described here has many advantages in terms of cost, time, and most importantly quantitation over the traditional plating assay and previously described quantitative approaches^8,10,14,51^. The end-point assay provides great flexibility for researchers in teaching labs or even high schools. With the end-point assay, a single plate reader can provide ample capacity for many students in a teaching lab to perform their own independent assays in parallel. Moreover, absorbance readers without incubation capabilities can be used because plates are cultured in standard incubators. Such readers are more commonly available than plate readers with incubating and multi-modal shaking, making this approach more widely available to a non-specialist lab. Furthermore, we expect this approach can be adapted even when a plate reader is not available. In this case, at the end-point, the contents of each well would be transferred to a cuvette and measured with a regular absorbance spectrophotometer. Finally, a high level of insight into phenomics can be obtained by combining the methods described here with previously described assays for monitoring viability using colony forming units and cell death using propidium iodine in yeast using the 96-well plate format^52,53^.

One of the main strengths of this approach is the use of IC_50_ as a quantitative read-out for growth inhibition. The IC_50_ is a more sensitive and accurate way to represent results than simply reporting changes in growth rate at a single chemical concentration, which is used in many other quantitative approaches^29,54,55^. Most notably, the use of a single chemical concentration can mask differences in sensitivity that are apparent using the IC_50_ approach. For example, with calcofluor white, the sensitivity of *ent3Δ/chs6Δ* was only apparent at concentrations of 50 µg/mL of CFW, however if this single concentration had been used, the differences between the remaining mutations would not have been observed because none of the other mutations showed appreciable growth at this concentration (Fig. 2A–C).

The steps for a successful adaptation of a traditional plating assay into a 96-well based IC_50_ assay are outlined in the Materials and Methods. One of the main considerations is the initial culturing conditions. It is important to obtain a uniform lawn of cells for accurate OD readings. To do this, we used a much higher starting cell concentrations than used by other published plate-reader based approach. Although a single starting concentration of cells worked for the diverse assays reported here, it may be necessary to optimize this starting concentration for different applications. However, as described in the materials and methods, such optimization is relatively simple, and should not preclude the application of this approach to diverse assays.

In addition to the cell concentration, we found that care was needed to keep the starting culture uniformly dispersed when adding chemicals to the wells. Without proper mixing at this step, the cells did not form a uniform layer. We found the best approach was to add at least 100 μL of chemical diluted in media to wells already containing the starting culture. Steady even mixing at this stage was important to obtain the uniform lawn needed for an accurate growth curve.

Despite its utility, this approach may not be appropriate for all chemicals. One concern is that, without shaking, cells may deplete oxygen or nutrients. We found that it was necessary to use at least 200 μL of culture in the well. With lower volumes, cells mounded in the center and edge of the well, likely due to the culture sloshing from side to side during reads (Fig. 1). This volume could lead to depletion of oxygen at the bottom of the well where the cells settle. While this did not seem to be an issue for the assays reported here, certain genotypes or chemicals may be incompatible with the static growth method described here. In such situations, pilot studies comparing the growth of cells in small agitated liquid culture to the static method will reveal whether an alternative approach is necessary. Furthermore, as noted with sertraline, for conditions where wild-type cells grow slowly, this method may be unable to capture minor increases in growth rate due to the shorter incubation times used. However, our successful adaptation of four disparate assays to the 96-well based IC_50_ assay format suggests that many chemicals will be amenable to this simple, inexpensive, and highly quantitative approach.

## Materials and Methods

### Yeast Strains and Plasmids

Yeast strains are listed in Table 3. Gene deletions were introduced by a standard PCR-based method^56^. Strains containing multiple genomic modifications were generated by standard yeast genetics.

**Table 3:** List of strains used.

Strain	Description	Source/Reference
SEY6210	*MATα leu2-3,112 ura3-52 his3-Δ200 trp1-Δ901 suc2-Δ9 lys2-801; GAL*	Robinson *et al*., 1988
SEY6211	*MATA leu2-3,112 ura3-52 his3-Δ200 trp1-Δ901 suc2-Δ9 lys2-801; GAL*	Robinson *et al*., 1988
DLY497	SEY6210 *ent5∆::TRP1, chs6Δ:TRP1*	Hung *et al*.^15^
DLY2349	SEY6210 *chs6d∆:TRP1, ent3dΔTRP1*	This Study
DLY2350	SEY6210 *chs6d∆:TRP1, ent3dΔTRP1*	This Study
DLY2351	SEY6210 *ent3∆::TRP1, chs6Δ:TRP1, ent5CR(R17E, R18E, K51E. H52E, L53E)::URA3*	This Study
DLY2352	SEY6210 *ent3∆::TRP1, chs6Δ:TRP1, ent5CB(D292A, L293A, I294A, D354A, L355A, I356A)::URA3*	This Study
DLY2353	SEY6210 *ent3∆::TRP1, chs6Δ:TRP1, ent5AB(E221A, F222A, E330A, F331A, F334A)::URA3-GFP-HIS3MX:URA3*	This Study
DLY2354	SEY6210 *ent3∆::TRP1, chs6Δ:TRP1, ent5ABCB(E221A, F222A, D292A, L293A, E330A, F331A, F334A, D354A, L355A, I356A)::URA3*	This Study
MDY421	SEY6211 *ENT5::URA3*	Hung *et al*.^15^
BY4742	*MATα his3Δ0 leu2Δ0 ura3Δ0 lys2Δ0*	Invitrogen
DLY742	*MATα his3Δ0 leu2Δ0 ura3Δ0 lys2Δ0 art1∆::KanMx*	Winzeler *et al*., 1999
DLY898	*MATa his3Δ0 leu2Δ0 ura3Δ0 lys2Δ0 art1∆::KanMx*	This Study
AJY1803	*MATa/α fpr1Δ/fpr1Δ tor1::tor1-1:URA3/tor1::tor1-1:URA3 MPS1-FRB-GFP:Kan/MPS1 ASK1-2xFKBP12:HIS3/ASK1*	Aravamudhan *et al*., 2005
AJY1807	*MATa/α fpr1Δ/fpr1Δ tor1::tor1-1:URA3/tor1::tor1-1:URA3 MPS1-FRB-GFP:Kan/MPS1 MTW1-2xFKBP12:HIS3/MTW1*	Aravamudhan *et al*., 2005
AJY2231	*MATa/α fpr1Δ/fpr1Δ tor1::tor1-1:URA3/tor1::tor1-1:URA3 MPS1-FRB-GFP:Kan/MPS1 DAD2/DAD2-2xFKBP12:HIS3*	Aravamudhan *et al*., 2005
AJY2231	*MATα fpr1ΔMPS1-1XFKBP12:HIS13 leu2Δ0::pHIS3-GFP-spc105(120*–*329) FRB:LEU2*	Aravamudhan *et al*., 2005
DLY2020	*MATα his3Δ0 leu2Δ0 ura3Δ0*	This study
DLY3090	*MATα his3Δ0 leu2Δ0 ura3Δ0 apm1∆::KanMx*	This study
DLY3091	*MATα his3Δ0 leu2Δ0 ura3Δ0 apm2∆::KanMx*	This study

### Media and Reagents

Yeast cells were grown in yeast/peptone media supplemented with 2% glucose and a mixture of adenine, uracil and tryptophan (YPD) or synthetic media supplemented with 2% glucose and an amino acid mix as previously described (SD)^57^. Calcofluor white fluorescent brighter, canavanine and rapamycin were obtained from Sigma. Sertraline and Myriocin were obtained from Fisher Scientific. Calcofluor white (10 mg/mL) and canavanine (2 mg/mL) stocks were prepared in water. Rapamycin (2 mg/mL), sertraline (10 mM) and myriocin (2 mM) stocks were prepared in DMSO.

### Replica pinning assay

To perform the replica pinning assay, 200 µL of log phase cells (OD_600_ = 0.5) were transferred into a well of a 96-well plate. The culture was serial diluted 5-fold in adjacent wells. The cells were replica pinned onto agar plates using a 48-well solid pin tool. Prior to use, the pin tool was sterilized by immersion of the pins into a 4 mm deep 5% bleach bath for 30 sec followed by immersion into a 70% ethanol bath for 30 sec followed by immersion into a 95% ethanol bath. The ethanol was removed by passing the pins through a flame to ignite the ethanol. The pin tool was immersed into the wells of the prepared 96-well plate and then gently pressed onto an agar plate without added chemicals. The replicator was then re-immersed without washing and gently pressed onto an agar plate with added chemicals. When multiple concentrations were used, the lowest chemical concentrations were pinned first. The plates were incubated at room temperature for 30 minutes to allow the liquid media to be absorbed into the agar and then the plates were transferred to an incubator.

### 96-well plate liquid culture assay

To perform the liquid culture assay, a log phase culture was diluted to 0.01 (OD_600_), as deterimined in a standard spectrophotometer, and transferred to a sterile reagent reservoir. For cells treated with calcofluor white, rapamycin, sertraline or myriocin cells were grown and diluted in YPD. For cells treated with canavanine, cells were grown and diluted in SD media supplemented. 100 µL of the diluted culture was distributed into the wells of a sterile 96-well assay plate using a multichannel pipette. Chemicals were added to the prepared 96-well plate as follows. Each chemical was diluted in media to a concentration twice the desired final concentration. The diluted chemicals were transferred to a standard deep-well 96-well plate. 100 µL of diluted chemicals were then transferred from the deep well plate to the corresponding wells of the prepared assay plate using a multichannel pipette and pipetted to mix three times.

Although, the conditions described worked well for the assays described, for different strain backgrounds and equipment, a pilot screen may be needed to determine the optimal starting cell concentration and chemical concentration. The ideal starting cell concentration results in a uniform lawn on the bottom of the well in untreated cells and a uniform albeit less dense lawn in treated wells where the cells grow. At the correct cell concentration, technical replicates will have identical growth curves. Cell concentrations that are too low will result in visible colonies and technical replicates will differ. To determine the optimal concentration for a new compound, we recommend starting with a 10-step, two-fold dilution series starting with twice the concentration reported for traditional plating assay. At the correct concentrations, the dilution series will capture the full dynamic range for both wild-type and mutants such that both will be unaffected at the lowest dose and (when possible) both strongly inhibited at the highest dose.

For continuous measurement of culture OD, an assay plate with lid was placed in a Spectra Max 340PC plate reader. Cells were incubated at 30 °C without shaking. Absorbance at 600 nm was collected every 30 min. It is not necessary to correct OD to 1 cm path-length. An accurate IC_50_ can be calculated as long as OD at the given time point is collected under the same condition. Alternatively, for end-point readings, an assay plate was placed with damp paper towels in a slightly ajar Styrofoam box within an incubator to protect the plate from desiccation. After 16–20 hours, plates were removed and OD measurements read. In cases where the uniform layer of cells were disturbed by moving them from the incubator, the cells were remixed prior to reading similarly to as previously described^25^. Briefly, the lid was removed from the 96-well plate and standard packing tape was applied to seal the wells. The plates were then vortexed gently to resuspend the cells and pelleted at 500 rpm for 10 sec in a swinging bucket centrifuge equipped with plate adaptors. The tape was removed prior to reading as described above.

### Optimization and Troubleshooting of 96 well assay

The following section describe challenges and solutions to adapt an existing plating based assay into a 96 wells plate assay to determine IC50 for chemicals of interest.

#### Obtaining uniform growth

In order to obtain a reliable reading for OD, cells must grow uniformly. There are several issues that can prevent uniform growth.

**Problem 1: Mounding**

**Figure 7:**
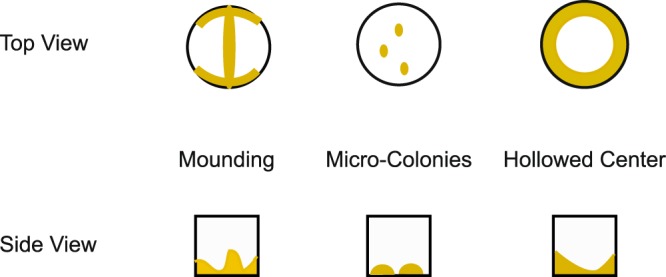
Schematic representation of potential problems in uniform growth. Uniform growth of a layer of cells is essential for reproducible OD readings. Several issues can impair uniform growth including (Left) mounding, of the cells in the middle or sides of the well. This can be caused by agitation or sloshing of the culture during reads. Solutions include omitting agitation, using a larger culture volume, or increasing the interval between reads. (Middle) Micro-colonies occurs when the cells are too dilute. Increasing the cell concentration will prevent the formation of microcolonies. (Right) Hollowed centers occur when chemical addition pushes the cells to the edges of the well. Using a larger volume of chemical and gentle thorough mixing will prevent hollowed centers.

**Cause 1:** Mounding occurs when the cells pile in the middle and accumulate on the edges. This can occur when a plate reader mixes on only one axis (Fig. 7).

**Solution**: During the time course, mixing should be disabled to avoid mounding. Instead, cells should be well mixed by pipetting before placing the 96-well plate into a plate reader.

**Cause 2:** In some cases the process of reading a plate can shake the plate enough to cause mounding even when mixing is not activated.

**Solutions**: To avoid this issue, increasing the volume within the well appears to have the most significant impact. However, reducing the reading frequency can reduce mounding as well.

**Problem 2: Micro-colonies**

**Causes**: Micro-colonies occur when the starting OD is too low to produce and even lawn (Fig. 7).

**Solution**: A pilot experiment should be performed with different starting concentrations of cells without any chemical. Each concentration should be run in at least 4 different wells to determine the cell concentrations that give reproducible growth cruves. We use 1x the minimal concentration of cells that gives a reproducible growth curve.

**Problem 3: Hollowed centers**

**Causes**: Hollowed centers occur when the cells are pushed out of the center of the well when chemical is added (Fig. 1).

**Solution**: To prevent hollowed centers, we fill each well with ½ the final volume of cells at 2x the final concentration. We then add the chemical to each well the remaining ½ final volume using a multi-channel pipette. We then gently mix the cells 6 times to ensure even distribution of cells.

**Problem 4: Evaporation**

**Causes**: The media in a 96 wells plate may evaporate when incubated at <30 °C over long period time due to high temperature and dry environment.

**Solution:** To overcome this issue, in a plate reader it may be necessary to drop the temperature. Alternatively, water can be added to the spaces between wells. In incubators evaporation can be avoided using a humid chamber that can prevent evaporation. We generally use a Styrofoam box containing 2–3 damp paper towels and place the entire box into the incubator.

#### Obtaining a full spectrum of inhibition

For a reliable IC50, the chemical concentrations tested must contain several concentrations with sub-maximal inhibition. The ideal dose dependent curve should have a sigmoidal shape.

**Approach:** We recommend starting with a 10-step, two-fold dilution series starting with twice the concentration reported for traditional plating assay using a sensitive and resistant strain. The IC50 is mainly calculated from data points at the linear range. Hence, researcher should use a wide range of chemical concentrations in order to correctly identify the linear range of the dose dependent curve.

#### Step 3: Choose the right media and growth condition

If a chemical has worked well in the traditional plating assay, but does not have the same effect using the 96-well assay there are several factors to consider.

**Problem 1: Chemical precipitation:** Some chemicals precipitate at high concentrations. This can obscure growth inhibition readings.

**Solution:** When using a new chemical for the first time, perform one experiment without cells to determine whether precipitation causes OD changes over time. If lower concentrations cannot be used, alternative media may reduce the problem (see below).

**Problem 2: Chemical inactivation:** Some chemicals are inactivated by media components. If a chemical works in YPD but not in synthetic media this is the most likely explanation.

**Solution:** Ammonium sulfate appears to be the most problematic component in commonly used media. If YPD cannot be used, mono sodium glutamate used as the nitrogen source may improve specific activity. Additional considerations are pH, the presence of chelating agents such as siderophorins or lectins in rich media.

**Problem 3: Aeration is necessary**. Assays requiring oxygenation may not be compatible with this approach.

**Solution:** To determine whether aeration is necessary, run a pilot experiment in an agitated liquid culture flask in parallel to the 96 well assays. If in the same media, effects are more pronounced in the agitated culture, the assay may not be compatible with 96-well adaptation.

**Problem 4: Competitive inhibitor in the media:** Chemicals such as toxic amino acids and glycolytic inhibitors can be out-competed by non-toxic counterparts from the media reducing their toxicity.

**Solution:** Whenever possible consider the mechanism of action and if possible exclude potential competitive inhibitors from the media.

**Problem 5: Growth inhibition of the vehicle:** Commonly used vehicles such as Ethanol and DMSO are themselves growth inhibitory.

**Solution:** The best practice is to use a uniform amount of vehicle in all wells. It is also advised that when running an experiment for the first time perform a vehicle control to assess the level of inhibition from the vehicle compared to no vehicle.

**Problem 5: Activation of general stress responses**. Yeasts have potent general stress responses activated by starvation, dehydration, irradiation and other insults. These stress responses can down-regulate transporters and cause chemical resistance. If the effects of chemicals are not pronounced in the 96-well assay, consider whether culturing conditions may have induced general stress responses.

#### Step 4: Avoid genetic drift or suppressors

Genetic drift due to inappropriate serial culturing can cause issues particularly with mutants that cause slow growth. It is best to avoid serial culturing and obtain fresh cells from frozen stocks routinely. In addition, screens should not be performed on cells previously subjected to chemical treatment as these may have acquired suppressor mutations.

### Half Maximal Inhibitory Concentration (IC50) Measurement

To calculate IC50 values from growth rate, the growth rate was determined as the slope of exponential growth phase using GraphPad Prism. The data were fit with the 4-parameter sigmoid curve:

$$y=No+\frac{{\rm{Nmax}}}{1+{e}^{(-{\rm{B}}({\rm{X}}-{\rm{X}}0))}},$$

where N_0_ is the average OD of the lag phase, Nmax is the average OD of the stationary phase, B is the slope of the log phase and X_0_ is the time point when log phase growth began. % growth rate was calculated by normalizing to the growth rate (B) of untreated controls. % growth rate was plotted it as the function of chemical concentration in log_10_ scale. IC50 values, the half way between the maximal and the minimal inhibition, were derived by a sigmoidal dose-response curve (variable slope, four parameters) using GraphPad Prism.

To calculate IC5_50_ values from OD, the time-point when wild type, untreated cells exited the exponential growth phase was determined. The OD from all wells at this time-point was recorded. % Growth was calculated by normalizing the absorbance reading of treated cells to the untreated control. % growth was plotted as the function of chemical concentration in log_10_ scale. IC50 values, the half way between the maximal and the minimal inhibition, were derived by a sigmoidal dose-response curve (variable slope, four parameters) using GraphPad Prism.

As an alternative approach IC50 values can be determined in Excel. To determine IC50, first a dose dependent curve is generated by plotting ercent of growth versus concentration in log_10_ scale. The data that falls outside of the linear range is omitted and the remaining data is fit with a natural log polynomial equation trend line. The equation of the trend line is: $y={\rm{A}}\ast \mathrm{ln}({\rm{x}})+{\rm{B}}$. IC50 is then determined by solving x, when y = 50.
